# Multiplexed Covalent Patterns on Double‐Reactive Porous Coating

**DOI:** 10.1002/asia.202200157

**Published:** 2022-04-13

**Authors:** Supriya Das, Ravi Kumar, Bingquan Yang, Sudipta Bag, Eric Sauter, Navid Hussain, Michael Hirtz, Uttam Manna

**Affiliations:** ^1^ Bio-Inspired Polymeric Materials Lab Department of Chemistry Indian Institute of Technology – Guwahati Kamrup Assam 781039 India; ^2^ Centre for Nanotechnology Indian Institute of Technology – Guwahati Kamrup Assam 781039 India; ^3^ Institute of Nanotechnology (INT) & Karlsruhe Nano Micro Facility (KNMFi) Karlsruhe Institute of Technology (KIT) 76344 Eggenstein–Leopoldshafen Germany; ^4^ Institute of Functional Interfaces (IFG) Karlsruhe Institute of Technology (KIT) 76344 Eggenstein–Leopoldshafen Germany; ^5^ School of Health Science and Technology Indian Institute of Technology – Guwahati Kamrup Assam 781039 India

**Keywords:** Porous Coating, Chemically Reactive, Pattern Interface, Dual Reactivity, 1,4-Conjugate Addition Reaction

## Abstract

We have conceptualized and demonstrated an approach based on the combination of hydrophobicity, a substrate‐independent dip coating as porous material with double residual chemical reactivities for implementing multiplexed, miniaturized and unclonable bulk‐infused patterns of different fluorophores following distinct reaction pathways. The embedded hydrophobicity (∼102°) restricted the unwanted spreading of beaded aqueous ink on the coating. The constructions of micropatterns on porous dip‐coating via ink‐jet printing or microchannel cantilever spotting offered orthogonal read‐out and remained readable even after removal of the exterior of the coating.

## Graphical abstract

**A double reactive**, porous and thick dip‐coating is rationally extended to introduce a complex, miniatured & three dimensional infused pattern‐following orthogonal chemical reactions with selected & representative two distinct fluorophores.

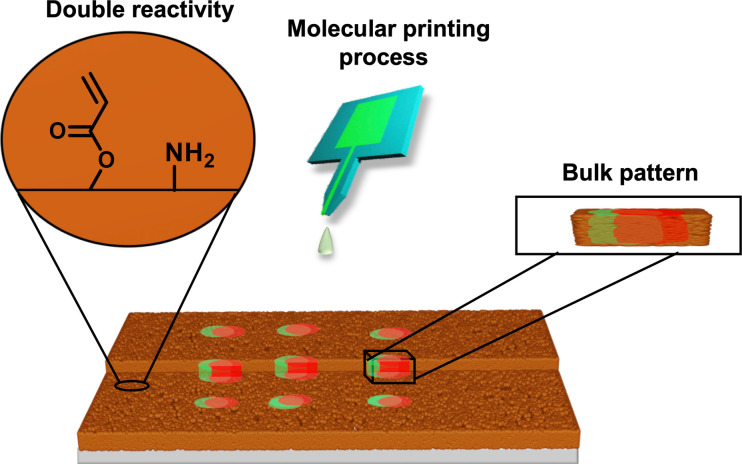

Spatially selective covalent modulation of ultrathin and chemically reactive coatings provides a simple basis to derive various functional patterned interfaces.[^1^, ^2^, ^3^, ^4^, ^5^] In the earlier reported patterned interfaces, mostly and commonly one type of residual reactivity was extended for spatially selective chemical modulation‐which is restricted to few nanometres across the used ultrathin coatings.[^1^, ^2^, ^3^, ^4^, ^5^] While the earlier reported patterns on ultrathin coatings remained inappropriate to sustain physical abrasion, the spatially selective integration of two distinct functional groups in the bulk of the coating following two independent chemical pathways would be interesting in both fundamental and applied contexts. For example, the dual modulation with desired chemistries at same location and orthogonal reading of complex pattern would be useful for high‐throughput and parallel screening of biomarkers, developing an effective anti‐counterfeiting interface, etc.[^6^, ^7^, ^8^, ^9^, ^10^] However, the utilization of a porous, inherently hydrophobic and dually chemically reactive interfaces for developing complex, bulk, unclonable and miniaturized patterned interfaces are rare in the literature.[^11^, ^12^]

Here, we utilized a dual chemically reactive porous dip coating (DCRPDC) inherently embedded with two distinct residual chemically reactive groups (acrylate and amine) for developing an orthogonally readable, abrasion tolerant and miniaturized bulk‐patterns through 1,4 conjugate addition reaction of the DCRPDC with selected fluorescent molecules at ambient conditions as shown in Scheme 1. The bulk chemical reactivity in the porous polymeric dip coating provided a basis to develop abrasion tolerant bulk‐pattern of selected small molecules. Moreover, the patterned interface remained washable due to the strategic association of robust covalent modifications. Further, the principle was successfully extended to develop QR codes using both microchannel cantilever spotting (μCS),[^6^, ^13^, ^14^] and ink‐jet printing process. Addition to the abilities of providing bulk‐modification, the dual chemical reactivity also allowed to associate two distinct florescent molecules following two independent chemical modulation processes. In the recent past, our lab utilized 1,4 conjugate addition reaction between selected reactants‐i. e. BPEI and 5Acl to develop different chemically reactive coating following different fabrication processes‐including layer‐by‐layer deposition technique, spray deposition etc. for associating the durable bio‐inspired liquid wettability.[^15^, ^16^, ^17^]

**Scheme 1:**
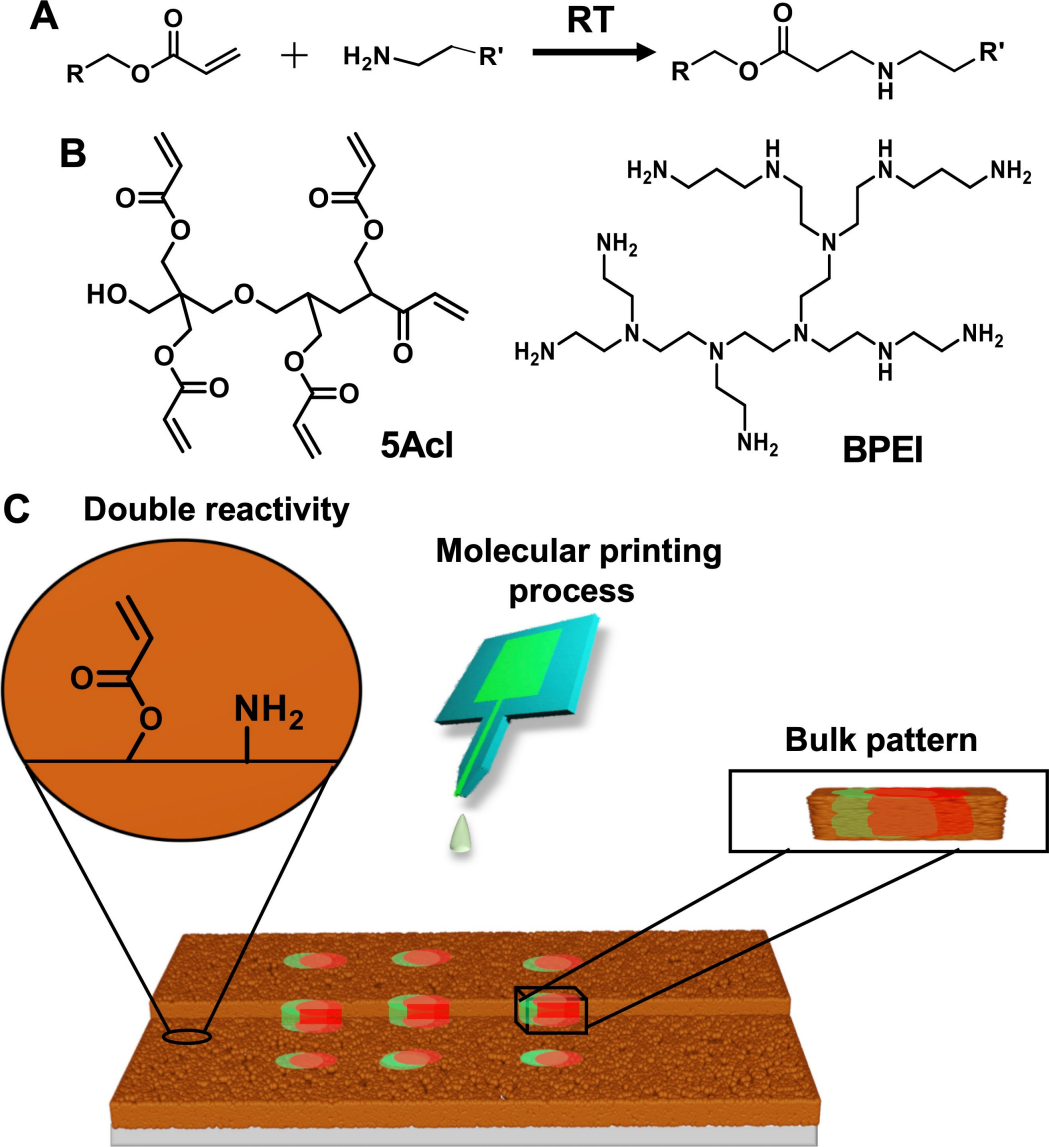
(A) Depicting Michael addition reaction between representative acrylate and amine groups. (B) Chemical structures of dipentaerythritol penta‐acrylate (5‐Acl) and branched polyethylenimine (BPEI). (C) Schematic representing a dual chemically reactive polymeric dip coating provided the spatially selective and bulk orthogonal‐covalent‐modification through molecular printing process.

The modulation of the single residual reactivity (i. e. acrylate group) that present in the highly rough (790±28 nm) and highly hydrophobic (with water contact angle (WCA) of ∼132°) dip‐coating (obtained with an immersion time of 10 minutes) allowed to embed bio‐inspired superhydrophobicity through rational post modification with primary amine containing selected hydrophobic (octadecylamine) small molecules.^[17]^ However, such a highly rough and hydrophobic interface would not be appropriate for creating a miniaturized patterned interface, as the transfer of aqueous ink from cantilever tip would be challenging. Therefore, in the current study, we introduced another chemically reactive dip‐coating by immersing the selected substrates (glass or paper) in the reaction mixture of BPEI/5Acl in 1‐heptanol for only 1 minute.

The prepared dip‐coating (thickness ∼3.2 μm) displayed moderate hydrophobicity with a WCA of ∼102° (Figure 1A). Moreover, the prepared dip‐coating was found to be significantly less rough (72±15 nm). The random deposition of the polymeric nanocomplex provided a porous morphology with aggregated granular microdomains (Figure 1B–D). The size and shape of the pores in the prepared coating is irregular with a wide range of distribution from ∼150 nm to ∼1 μm. Another advantage of the current coating is the existence of two distinct residual chemically reactivities‐i. e. amine and acrylate (Scheme 1C). The ATR‐FTIR analysis of the dip‐coating confirmed the presence of residual acrylate groups where the characteristic IR peaks for C−H deformation of vinyl groups and ester carbonyl stretching appeared at 1408 cm^−1^ and 1733 cm^−1^, respectively, as shown in Figure 1E (black). An additional experiment was designed to examine the existence of residual acrylate groups in both the surface and bulk of the prepared dip‐coating. The coating was physically abraded by applying adhesive tape peeling process and the successive application of adhesive tape gradually lowered its thickness (from 3.2 μm to 1.6 μm) and randomly exposed the interior of the dip‐coating. Hardly any change in FTIR signature of residual acrylate groups was noted in the dip‐coating before and after incurring the physical abrasion process as shown in Figure 1E. The intensity of the normalized (with respect to ester carbonyl stretching at 1733 cm^−1^) and the characteristic IR signature for C−H deformation of the β carbon of the vinyl moiety remained unaltered before and after the random exposure of the interior of the coating. The freshly exposed interior of the physically abraded coating revealed the presence of porous features (Figure S1). As expected, the sand‐paper abraded interface was also found to exhibit residual acrylate groups (Figure S2). On the other side, the XPS signature of N 1s at ∼400 eV revealed the presence of residual amine (Figure S3) in the prepared dip‐coating. Such prepared dip‐coating can be successfully applied on plastic, metal, ceramic and wood (See Figure S4).

**Figure 1:**
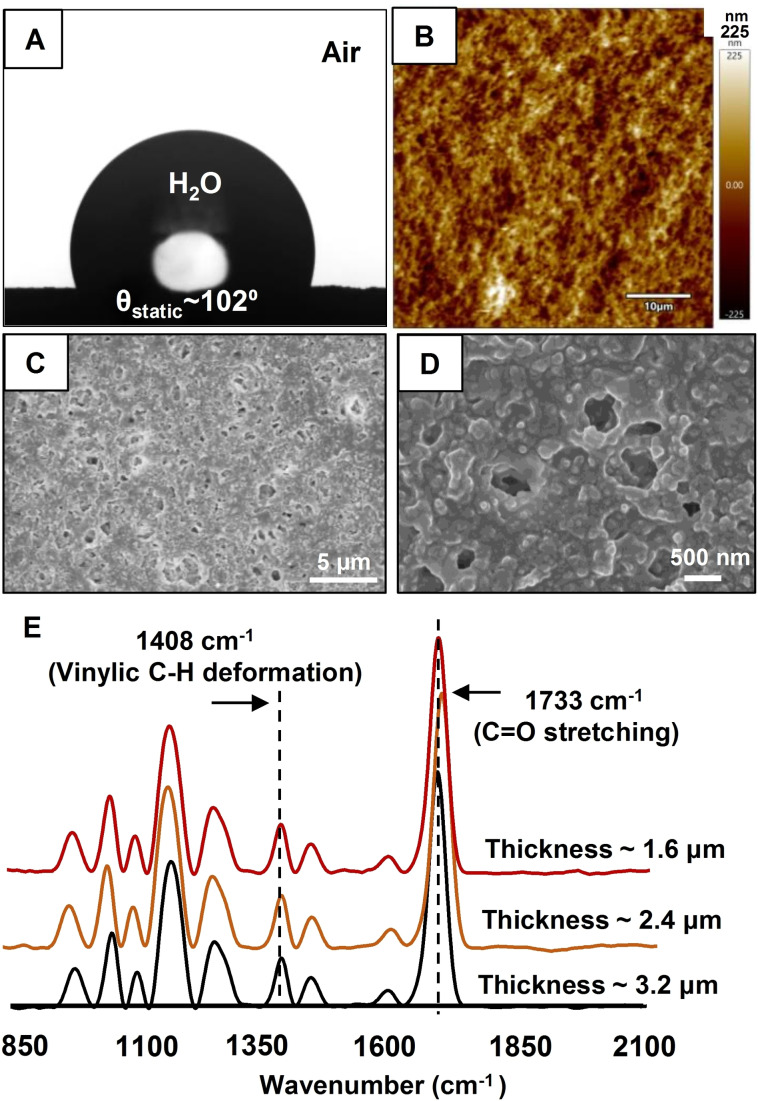
**(**A) The contact angle image of beaded water droplet on the polymeric dip coating. (B) AFM and (C–D) FESEM image of the polymeric dip‐coating in (C) low and (D) high magnifications. (E) ATR‐FTIR spectra accounting the presence of residual acrylate group in the synthesized dip‐coating before (black) and after (orange and red) exposure of the interiors.

Next, the existence of dual chemical reactivity in the prepared dip‐coating was characterized by microscopic imaging and FTIR spectral analysis. First of all, two aqueous droplets of two distinct fluorophores, i. e. tetramethylrhodamine (TMR; non‐reactive) and tetramethylrhodamine cadaverine (TMRC; readily reactive due to the presence of primary amine moiety) were manually beaded on the prepared dip‐coating. As expected, two circular spots were observed in the red channel in fluorescence microscopy (ESI Figure S5A–C). After the DI water and followed by ethanol washing, the circular spot made of TMRC remained unaffected. However, the other spot formed out of TMR disappeared (ESI Figure S5D). While the 1,4 conjugate addition reaction between the primary amine and residual acrylate of the dip‐coating allowed a covalent immobilization of TMRC (Figure 2A), the lack of such available primary amine in TMR failed – as expected – to provide covalent association with the dip‐coating. Rather the physically deposited TMR readily washed away after the application of the DI water and ethanol washing process. In another experiment, the aqueous droplet of same TMRC beaded on the hexylamine modified dip‐coating failed to survive such washing treatment as shown in ESI Figure S5E–F. This simple demonstration validated that the residual acrylate groups remained highly reactive to primary amine containing small molecules and provide a facile basis for covalent immobilization of selected molecules at ambient condition. On the other side, the same dip‐coating after manual deposition of two distinct aqueous droplets of fluorescein isothiocyanate (FITC, ESI Figure S5A) and fluorescein (Figure S1B) displayed two distinct circular and fluorescent spots under the fluorescence microscope as shown in Figure S5G. However, the regular washing of the dip‐coating with DI water and ethanol resulted in the disappearance of the fluorescein based circular spot, while the FITC derived spot remained unaffected under same treatment (ESI Figure S5H), due to the covalent attachment of FITC with residual amine of the dip‐coating (Figure 2A). In contrast, the dip‐coating pre‐modified with butyl‐acrylate failed to provide such covalent attachment to the FITC due to lack of residual amine, and thus the deposited FITC readily washed off on DI water exposure as shown in Figure S5I–J. Thereafter, the post‐modifications of the dip‐coating with FITC and TMRC were also characterized with ATR‐FTIR spectral analysis as shown in Figure 2B. The post modification of the dip‐coating with TMRC resulted in the depletion of normalized IR peak intensity for C−H deformation of the vinyl group at 1408 cm^−1^ with respect to the carbonyl stretching at 1733 cm^−1^. The depletion of the IR peak intensity unambiguously supported the 1,4‐conjugate addition reaction between the primary amine of the TMRC and the residual acrylate of the dip‐coating, where the vinyl moiety of the acrylate is compromised while the carbonyl group remained intact. On the other hand, the residual amine of the dip‐coating remained highly reactive towards thiocyanide groups. The same coating that was exposed to FITC provided a characteristic IR signature for the N−C=S band at 1570 cm^−1^ as shown in Figure 2B (green). Thus, both the residual groups‐acrylate and amine‐remained chemically reactive towards both the amine of TMRC and the thiocyanide of FITC, respectively. Furthermore, the confocal microscopy imaging confirmed the bulk immobilization of both TMRC and FITC on the chemically reactive dip‐coating as shown in ESI Figure S6 and Figure 2C–D. The merged (both green and red channel) confocal image in Figure 2E revealed the existence of a common section (indicated by yellow color) that is modified with both, TMRC and FITC. Such strategic dual modifications of the chemically reactive interface with two distinct representative fluorophores following two independent reaction pathways allowed to develop a complex luminescent pattern. On the other side, the bulk chemical modifications of the dip‐coating with selective fluorophores provided an abrasion tolerant patterned interface (Figure 2E–F), where the same patterned interface continued to display the desired fluorescence signal‐even after the physical abrasion as shown in Figure 2F. In a separate experiment, the presence of red‐stain in the physically abraded interface validated the bulk diffusion of used small molecule (TMRC, Figure S7). The prepared polymeric coating on other substrate also remained efficient to sustain repetitive adhesive tape peeling test as shown in Figure S8. Encouraged by the multi‐functional and abrasive resistant chemical modifications established, trials for miniaturized and functional patterns were implemented. For this task, inkjet printing and microchannel cantilever spotting (μCS) were employed. In μCS, inks are spotted in an atomic force microscopy (AFM) by a cantilever with a microchannel connected to an on‐chip reservoir for μL volumes of ink, allowing for chemical reactions in such “micro‐reactors”.[^4^, ^18^, ^19^] When the cantilever is brought into contact with a surface, ink can transfer by capillary force from the reservoir to the surface. On porous and moderately hydrophobic substrates, this allows for delivery of sufficient volume of ink for imbibition and bulk functionalization of the substrate interior.[^20^, ^21^, ^22^] With the highly‐precise and reproducible position control over the cantilever, arbitrary patterns can be formed and even subsequent deposition of different inks onto the same spot can be achieved.^[6]^ First, as example of arbitrary micropatterns, letters “IIT” and “CNF” were spotted with TMRC and FITC inks via μCS (Figure 3). Figure 3A and B show the single channel fluorescence microscopy images for each ink, which – when combined – reveal an overlap of both patterns demonstrating the dual functionalization of the surface also on the micronscale (Figure 3C). This multiplexing of inks also allows for selective readout of information by selection of the matching filter channel to reveal different information from the same pattern. Importantly, the micropatterns even stay legible after abrasion (Figure 3D–E), enabling a robust information storage. Further, a QR code pattern was printed by inkjet printing FITC ink onto a dip‐coated paper (Figure S9A). The fluorescence imaging confirmed the development of a QR code (ESI Figure S9A) capable of sustaining physical abrasion. The interface was physically abraded through adhesive tape peeling as evident from the confocal image of the QR code before and after abrasion as shown in ESI Figure S9C–D. To clearly show the impact of the abrasion treatment to the QR code pattern, fluorescence images were obtained under same exposure conditions and also at an exposure time allowing for the full dynamic range to be covered. Though a slight overexposure would actually benefit direct read‐out of the barcode from such an image e. g. by mobile phone, a simple contrast enhancement allows even the abraded QR code to be reliably readout directly (ESI Figure S10). Another important aspect of the current approach is the association of unclonable features in the miniatured patterns‐which is important for anticounterfeiting applications.^[7]^ Here, the random fluctuation caused uncontrollable during the patterning could act as an unclonable fingerprint – which cannot be achieved on smooth interface and uncoated paper (ESI Figure S11).

**Figure 2:**
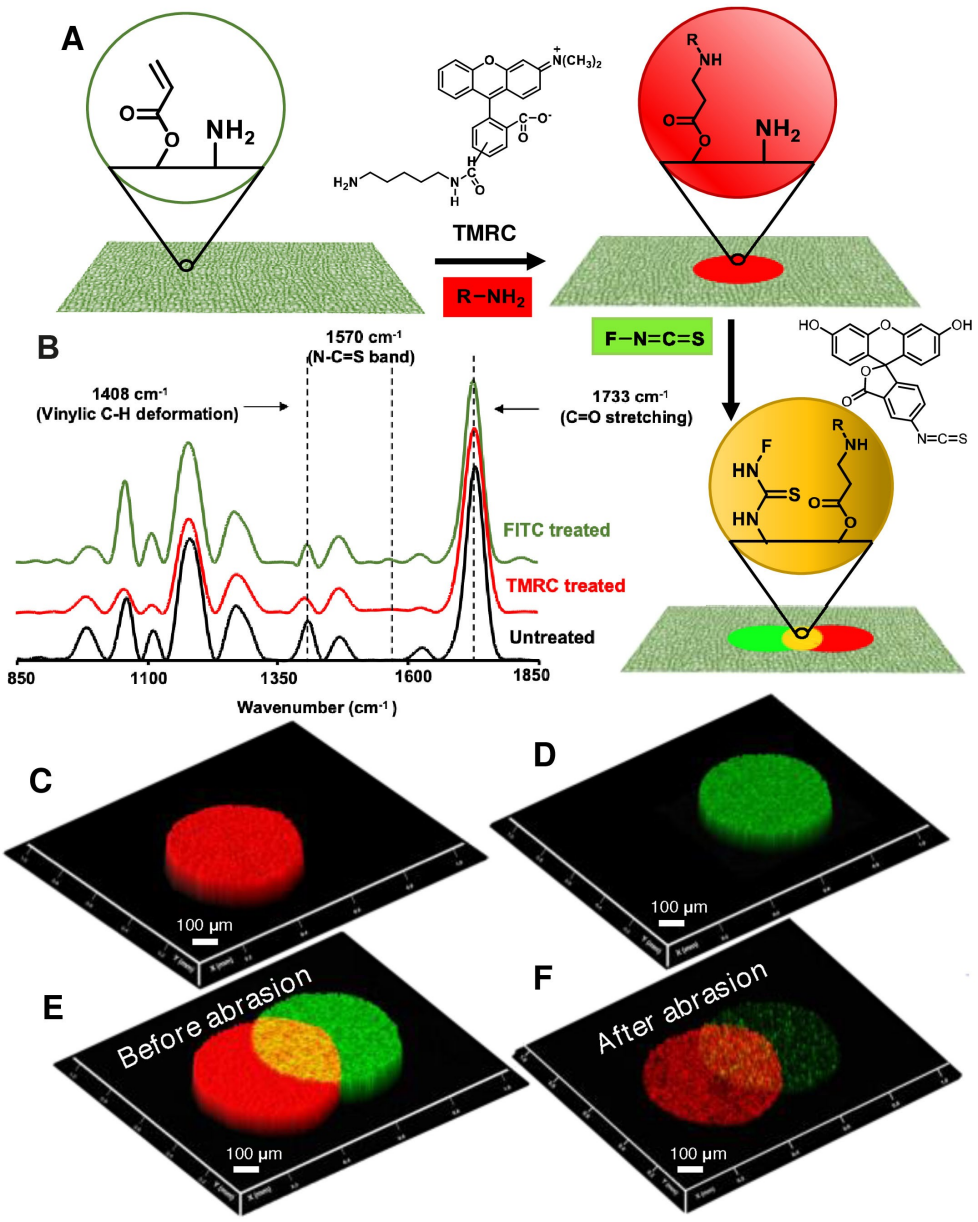
(A) Schematic illustrating the covalent and spatially selective modifications of dual chemically reactive dip‐coating with two distinct fluorescent inks (tetramethylrhodamine cadaverine (TMRC) and fluorescein isothiocyanate (FITC), where TMRC and FITC mutually reacted with residual acrylate and amine respectively at ambient condition. (B) ATR‐FTIR spectra accounting the dual chemical modification of dip‐coating with TMRC and FITC. (C–D) The confocal microscopic images revealed the presence of bulk patterns of TMRC (C) and FITC (D), respectively. (E–F) Merged confocal images of the patterned interface before (E) and after (F) physical abrasion.

**Figure 3:**
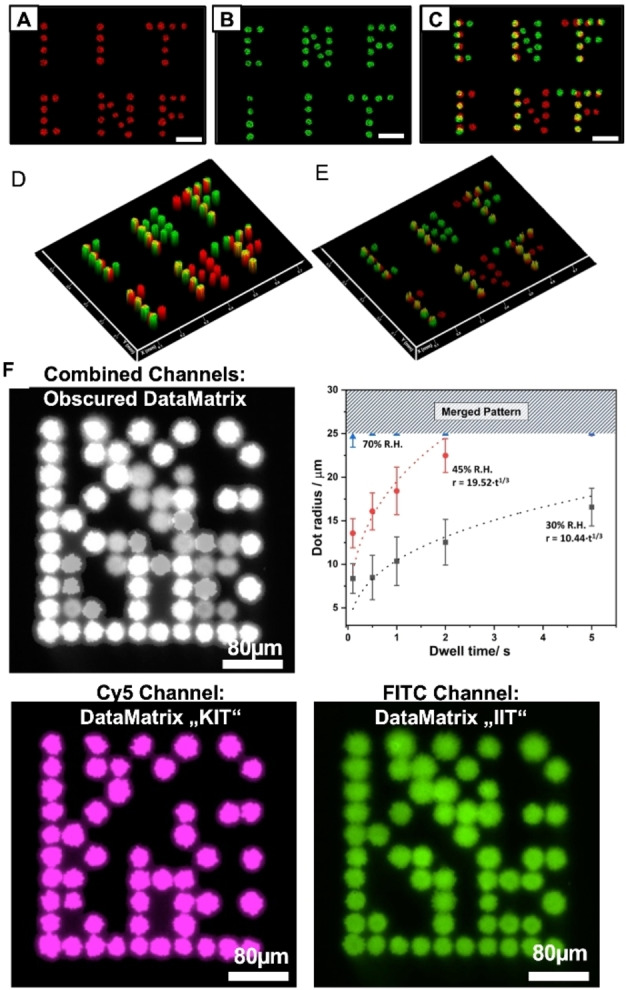
Functional and abrasive resistant micropatterns. Confocal microscopy images (scale bar 100 μm) of (A) TMRC and (B) FITC fluorescence channel of μCS spotted letters. A combined image of the fluorescence channels in (C) shows the overlap between the patterns (D) before and (E) after abrasion. (F) Combined and single channel fluorescent microscopy images of two different DataMatrix codes printed one over the other with different fluorophore inks. While the combined channel obscures the information, the single channels reveal readable codes. The images are intentionally slightly overexposed for easier readout of the code. The graph shows the radius of features spotted with different dwell times and at different relative humidity (R.H.) during the patterning process, source images are given in ESI Figure S13.

The presented porous and dip‐coating allows for a realization of such unclonable features, in particular for the smaller sized features within micropatterns (Figure 3F). While bigger spot features as e. g. the manually spotted features in the size range of several hundred show a rather well‐defined delineation, the smaller features from μCS approach offer more rugged borders as of the intrinsic randomness of the pores in the substrate becoming visible on this scale of patterning (ESI Figure S12).

For a demonstration, two different DataMatrix patterns were spotted via μCS (Figure 3F). When both channels are overlayed (implying readout without proper fluorescence filtering), the DataMatrix is obfuscated and cannot be read. The single channels as orthogonal readout enable proper recognition. As mentioned above, a proper unclonable feature border is in particular obvious in smaller features, which fortunately are readily available via μCS and can be even tuned via dwell time (tip/surface contact time) and relative humidity under which the spotting takes place (Figure 3F, ESI Figure S13). Theory predicts, imbibition of a fluid into a porous substrate should follow a dependency on time proportional to t^1/3^.^[26]^ This was also shown to be the case for other porous coatings in μCS and our current results are in line with this mode of diffusive imbibition of ink into porous substrates.[^20^, ^21^] The imbibition rate becomes higher with higher humidities during spotting reflecting the more hydrated state of substrate and ink at raised humidity enabling a faster flow. As the centre distance between features in the test array was arbitrarily set to 50 μm, the dots features start to merge after reaching a feature radius of 25 μm, indicated by the hatched area in the top of the graph. Below this restraint, feature radius size could be tuned down to (7.8±1.7) μm by lowering humidity and dwell time. Generally, for lowest dwell time (0.1 s) the radius is a bit larger than what is expected from the theoretical description. This can be understood as a small additional amount of ink that will snap off from a meniscus that builds on retraction of the μCS tip and continue to be adsorbed into the substrate. This snap off volume will be similar for each dwell time, therefore affecting feature size in particular for the smallest dwell times where the imbibed volume directly from the tip is also smallest.

The granularity of the coating is on the length scale of about 500 nm, as seen in Figure 1B–D. On the one hand, this limits the maximal accuracy in patterning to about 1 to 2 μm (as seen in the standard deviation of the smallest obtained features being ±1.7 μm), but on the other hand enables an intrinsic anti‐counterfeiting approach based on the borders of the spots.

While the general readout of the DataMatrix micropattern is facilitated by a slight overexposure of the fluorescence images, images preserving the fully dynamic range of the fluorescence signal are in particular well suited for observing the unclonable features of the spot delineation (Figure S14). Even by inspection with naked eye, the borders of the dots already reveal obviously distinct and highly recognizable shapes. The use of machine vision and artificial intelligence could readily enable automated recognition and identification of such features for regular applications,[^23^, ^24^, ^25^] while the intrinsic randomness of the porous substrate ensures uniqueness and prohibits cloning. The patterns remain stable also under water with no observable swelling or deterioration of the coating or pattern, thus providing a reliable non‐clonable feature (Figure S15).

In summary, we report a dual chemically reactive and porous dip‐coating for depicting bulk and complex pattern that enabled orthogonal read out of two distinct patterns in parallel. Further, the strategic post covalent modification with various types of selected and appropriate molecules, proteins, DNA or nanoparticles would allow to develop a different functional material for practically relevant applications including sensing, anticounterfeiting etc. In future, we will explore such chemically reactive porous polymeric coating in developing rewritable patterns.

## Conflict of interest

The authors declare no conflict of interest.

## Supporting information

As a service to our authors and readers, this journal provides supporting information supplied by the authors. Such materials are peer reviewed and may be re‐organized for online delivery, but are not copy‐edited or typeset. Technical support issues arising from supporting information (other than missing files) should be addressed to the authors.

Supporting Information

## Data Availability

The data that support the findings of this study are available from the corresponding author upon reasonable request.

## References

[1] F. Bally , L. Gall , C. Hussal , J. Kramer , K. Cheng , R. Kumar , T. Eyster , A. Baek , V. Trouillet , M. Nieger , S. Bräse , J. Lahann , Chem. A Eur. J. 2017, 23, 13342. 10.1002/chem.201700901 28644514

[2] F. Bally , K. Cheng , H. Nandivada , X. Deng , A. M. Ross , A. Panades , J. Lahann , ACS Appl. Mater. Interfaces 2013, 5, 9262. 23888837 10.1021/am401875x

[3] C. Mawélé Loudy , J. Allouche , A. Bousquet , H. Martinez , L. Billon , Nanoscale 2020, 12, 7532. 32219294 10.1039/c9nr10740a

[4] S. M. M. Dadfar , S. Sekula-Neuner , U. Bog , V. Trouillet , M. Hirtz , Small 2018, 14, 1800131. 10.1002/smll.201800131 29682874

[5] F. Behboodi-Sadabad , H. Zhang , V. Trouillet , A. Welle , N. Plumeré , P. A. Levkin , Adv. Funct. Mater. 2017, 27, 1700127.

[6] J. Atwater , D. S. Mattes , B. Streit , C. von Bojničić-Kninski , F. F. Loeffler , F. Breitling , H. Fuchs , M. Hirtz , Adv. Mater. 2018, 30, 1801632. 10.1002/adma.201801632 29938845

[7] T. Ma , T. Li , L. Zhou , X. Ma , J. Yin , X. Jiang , Nat. Commun. 2020, 11, 1811. 32286298 10.1038/s41467-020-15600-6 PMC7156701

[8] J. F. C. B. Ramalho , S. F. H. Correia , L. Fu , L. L. F. António , C. D. S. Brites , P. S. André , R. A. S. Ferreira , L. D. Carlos , Adv. Sci. 2019, 6, 1900950. 10.1002/advs.201900950 PMC6774024 31592146

[9] A. Khlyustova , Y. Cheng , R. Yang , J. Mater. Chem. B 2020, 8, 6588. 32756662 10.1039/d0tb00681e PMC7429282

[10] M. Benz , A. Asperger , M. Hamester , A. Welle , S. Heissler , P. A. Levkin , Nat. Commun. 2020, 11, 5391. 33106489 10.1038/s41467-020-19040-0 PMC7589500

[11] R. Arppe , T. J. Sørensen , Nat. Chem. Rev. 2017, 1, 0031.

[12] S. Canossa , S. Wuttke , Adv. Funct. Mater. 2020, 30, 2003875.

[13] J. Xu , M. Lynch , J. L. Huff , C. Mosher , S. Vengasandra , G. Ding , E. Henderson , Biomed. Microdevices 2004, 6, 117. 15320633 10.1023/b:bmmd.0000031748.13353.10

[14] J. Xu , M. Lynch , S. Nettikadan , C. Mosher , S. Vegasandra , E. Henderson , Sens. Actuators B 2006, 113, 1034.

[15] D. Parbat , U. Manna , Chem. Sci. 2017, 8, 6092. 28989639 10.1039/c7sc01055a PMC5625591

[16] K. Maji , U. Manna , J. Mater. Chem. A 2018, 6, 6642.

[17] S. Das , A. Das , D. Parbat , U. Manna , ACS Appl. Mater. Interfaces 2019, 11, 34316. 31429551 10.1021/acsami.9b11113

[18] S. M. M. Dadfar , S. Sekula-Neuner , V. Trouillet , M. Hirtz , Adv. Mater. Interfaces 2018, 5, 1801343. 10.1002/smll.201800131 29682874

[19] S. M. M. Dadfar , S. Sekula-Neuner , V. Trouillet , M. Hirtz , Adv. Mater. Interfaces 2021, 8, 2002117.

[20] M. Hirtz , M. Lyon , W. Feng , A. E. Holmes , H. Fuchs , P. A. Levkin , Beilstein J. Nanotechnol. 2013, 4, 377. 23844343 10.3762/bjnano.4.44 PMC3701425

[21] M. Hirtz , W. Feng , H. Fuchs , P. A. Levkin , Adv. Mater. Interfaces 2016, 3, 1500469.

[22] G. Arrabito , V. Ferrara , A. Ottaviani , F. Cavaleri , S. Cubisino , P. Cancemi , Y. P. Ho , B. R. Knudsen , M. S. Hede , C. Pellerito , A. Desideri , S. Feo , B. Pignataro , Langmuir 2019, 35, 17156. 31790261 10.1021/acs.langmuir.9b02893

[23] X. He , Y. Gu , B. Yu , Z. Liu , K. Zhu , N. Wu , X. Zhao , Y. Wei , J. Zhou , Y. Song , J. Mater. Chem. C 2019, 7, 14069.

[24] Y. Liu , F. Han , F. Li , Y. Zhao , M. Chen , Z. Xu , X. Zheng , H. Hu , J. Yao , T. Guo , W. Lin , Y. Zheng , B. You , P. Liu , Y. Li , L. Qian , Nat. Commun. 2019, 10, 2409. 31160579 10.1038/s41467-019-10406-7 PMC6547729

[25] H. Im , J. Yoon , J. Choi , J. Kim , S. Baek , D. H. Park , W. Park , S. Kim , Adv. Mater. 2021, 33, 2102542. 10.1002/adma.202102542 34514649

[26] J. Xiao , H. A. Stone , D. Attinger , Langmuir 2012, 28, 4208. 22283599 10.1021/la204474f

