# Supplementary material for: Multiplexed Covalent Patterns on Double‐Reactive Porous Coating
Source: Chem Asian J. 2022 Apr 13;17(11):e202200157. doi: 10.1002/asia.202200157 (PMC9324105; doi:10.1002/asia.202200157)
Supplement: Supplementary file 1 — Supporting Information [file ASIA-17-0-s001.pdf]

CHEMISTRY
AN ASIAN JOURNAL

Supporting Information

Multiplexed Covalent Patterns on Double-Reactive Porous Coating

Supriya Das, Ravi Kumar, Bingquan Yang, Sudipta Bag, Eric Sauter, Navid Hussain, Michael Hirtz,* and Uttam Manna*© 2022 The Authors. Chemistry - An Asian Journal published by Wiley-VCH GmbH. This is an open access article under the terms of the Creative Commons Attribution License, which permits use, distribution and reproduction in any medium, provided the original work is properly cited.

Experimental Section:

1. Materials. Branched poly(ethyleneimine) (BPEI, MW ~25000 Da), dipentaerythritol pentaacrylate (5Acl, MW ~524.21 g mol^{-1}), tetramethylrhodamine cadaverine (TMRC) and fluorescein isothiocyanate (FITC) were purchased from Sigma-Aldrich, Bangalore, India. Methanol was purchased from EMPLURA, Mumbai, India. Ethanol was purchased from TEDIA, USA. 1-heptanol was procured from Alfa Aesar. THF was purchased from RANKEM, Maharashtra, India. Microscopic glass slides were obtained from JSGW (Jain Scientific Glass Works, India). Adhesive tape (Johnson tape Ltd. India) was purchased from local sources. Rhodamine-6G and fluorescein were acquired from LOBA Chemie (Laboratory Reagents and Fine Chemical) Mumbai, India.

2. Preparation of the Randomly Aggregated Thin Polymeric Layer. 5Acl (265 mg mL^{-1}) and branched polyethylenimine (BPEI, 50 mg mL^{-1}) solutions in 1-heptanol were first prepared in two separate glass vials. Then, 1.2 mL of BPEI solution was mixed with 4 mL of 5Acl solution to prepare reaction mixture, and thereafter selected substrates a clean glass slide or paper was dipped into the reaction mixture. The substrate was kept into the solution for 1 min. After that, the coated substrate was taken out of the reaction mixture and dried in air, followed by thorough washing with THF.

3. Surface Characterization.

3.1 Contact Angle Measurements. Contact angles of beaded water droplets (5 μL) were measured using a KRUSS Drop Shape analyser-DSA25 instrument with an automatic liquid dispenser at ambient temperatures. The static water contact angles were measured at four different locations on the prepared coating to calculate standard deviation as a measure of error. We have applied sessile drop method to acquire the contact angle.

3.2 Scanning Electron Microscopy (SEM). All the samples were coated with a thin layer of gold sputter prior to obtaining SEM images of the dip-coatings using a Carl Zeiss Field Emission Scanning Electron Microscope (FESEM). The images were acquired in InLens mode. Image J software was used to measure surface pore-size from FESEM images.

3.3 Infrared Spectroscopy. ATR-IR spectra were recorded using Spectrum Two UATR, PerkinElmer at an ambient temperature with single reflection mode, Spectrum 10 software was used to process the data. The instrument pressure arm was used to achieve improved contact between the modified and unmodified coatings and the crystal, prior to record the data. The associated software (Spectrum 10 software) of the equipment was used for smoothening the data with smoothening factor of 25.

3.4 Atomic Force Microscopy (AFM). AFM images was acquired on a Oxford Instrument, MFP-3D Origin system in tapping mode with silicon tip (AC160TS-R3, nominal frequency 325 kHz, nominal force constant 40 N/m). Three different regions of the prepared dip-coating were analyzed.

3.5 Optical Imaging. The digital images were captured by using Nikon Coolpix b700 digital camera. Thickness of the material was measured using stylus surface profilometer (Veeco-Dektak 150). Bright field, fluorescence microscopic images and z-stacking images were captured by using a ZEISS Axio Vert.A1 inverted microscope and Zeiss LSM 880 Laser Scanning Confocal Microscope. Additional fluorescence microscopy for the micropatterns was performed on a Nikon Eclipse 80i upright fluorescence microscope (Nikon, Germany) equipped with an Intensilight illumination (Nikon, Germany), a Nikon DS Qi2 camera, and Cy5 and FITC filters (Nikon Y-2E/C). The spot diameters were measured by the built-in NIS-element software (Nikon, Germany) on the microscope for the images presented in ESI Fig. S6. The S.D. from these diameters was reported as error.

3.6 X-ray photoelectron spectroscopy (XPS) measurements. The X-ray photoelectron spectroscopy (XPS) measurements were carried out under an ultra-high vacuum conditions with a base pressure of 1×10^{-9} mbar. Core-level spectra were recorded under normal emission with a Scienta R4000 hemispherical electron analyzer using Al-Kα radiation (1486.6 eV). Firstly, for every sample the survey XPS spectrum was measured and no unexpected contaminations were observed in these spectra. For a precise determination of the N 1s lines position and necessary correction the XPS Peak 41 software was used.

4. Fabrication of Spatially Selective Pattern Interfaces. Chemically reactive porous and moderately rough interfaces were separately and manually exposed to aqueous droplet of TMRC as well as FITC respectively to develop luminescent circular spot on the dip-coating. The substrate was thoroughly washed with ethanol and DI-water to remove loose bound and unreacted TMRC and FITC. The larger QR-Codes (Fig. 3) were obtained by printing an aqueous solution of FITC (concentration of 0.1 mg/ml) with a commercial consumer grade inkjet printer (Canon PIXMA G2021) with printing resolution of 4800 × 1200 dpi onto dip-coated paper. The initial micropatterns (Fig. 3) were spotted via μCS on a Nano eNabler System (Bioforce Nanosciences). Firstly, the microchannel-cantilever was cleaned by ozone treatment for 5 minutes. Then, the selected ink, i.e., aqueous solution of tetramethylrhodamine cadaverine (TMRC, 0.1mg/ml) was loaded to microchannel cantilever (SPT-S-30, Bioforce Nanosciences), and the cantilever was adjusted systematically to touch only the upper periphery of the substrate (to avoid spillages of the ink) for delivering the ink solution to the dip-coating. The written pattern using TMRC was prepared on the dip-coated hydrophobic polymeric substrate which was previously manually marked with a marker to maintain the design of the pattern along a fixed line and position and used as a reference for the next superimposed pattern prepared by fluorescein isothiocyanate (FITC, 0.1 mg/ml) on that substrate after the air drying of the previously prepared micro-patterns. Then the whole patterned substrate was placed under laser scanning confocal microscope to capture the fluorescence images. The miniaturized DataMatrix Codes (Fig. 3) and spot size trials (ESI Fig. S6) were spotted by μCS on a NLP 2000 instrument (Nanoink, Inc.). The microchannel-cantilever (SPT-S-C10S) was purchased from Bioforce Nanosciences. Prior to use, the microchannel-cantilever was plasma-cleaned by oxygen plasma (0.2 mbar, 100 W, 20 sscm O_2, 2 min) on a Diener plasma system Atto. Streptavidin_alexa 647 (ThermoFisher Scientific, Germany, 1 μg μL^{-1} in DMSO, magenta channel) and FITC (Sigma-Aldrich, Germany, 1 μg μL^{-1} in DMSO, green channel) were used as fluorescent dyes. The microchannel-cantilever reservoir was then filled with 0.5 μL of ink, and the ink was pushed into the pen by blowing with a nitrogen stream. All patterning was done at room temperature, with control humidity and dwell time. All DataMatrix patterns were printed at 30% RH and 0.5 S of dwell time. The dot arrays were printed at different humidity (20%, 45%, 70% RH) and dwell time (0.1, 0.5, 1, 2, 5 sec). The DataMatrix and dot arrays were designed by in-built software in NLP 2000 instrument. After printing, the samples were washed immediately with DI water (18.2 MΩ cm, Arium water system, Sartorius, Germany) to make sure to remove unbound and excess ink. The samples were then dried with nitrogen before further analysis by optical microscopy.

5. Abrasion Test. For abrasion tests, a double-sided adhesive tape (1 × 1 cm) was first attached onto a microscopic glass slide, and then, the patterned dip coated substrate (3 × 1 cm) was brought in contact to the adhesive tape with an applied load of 500 g. The external load is applied to facilitate a uniform and homogeneous contact between the substrate and the tape. After 20 min, the adhesive tape was peeled off of the substrate. After removal of the adhesive surface, it was found that top portion of the coating was transferred to that adhesive surface partially, and the interiors of the coating was arbitrarily exposed. Fluorescent patterns were observed to be readable even after 25 cycles of adhesive tape peeling test. To perform sand paper abrasion test, an abrasive sand paper with 4 cm length and 2.5 cm width was exposed to freshly prepared coating with external load of 100 g and applied a back and forth motion for 25 times.

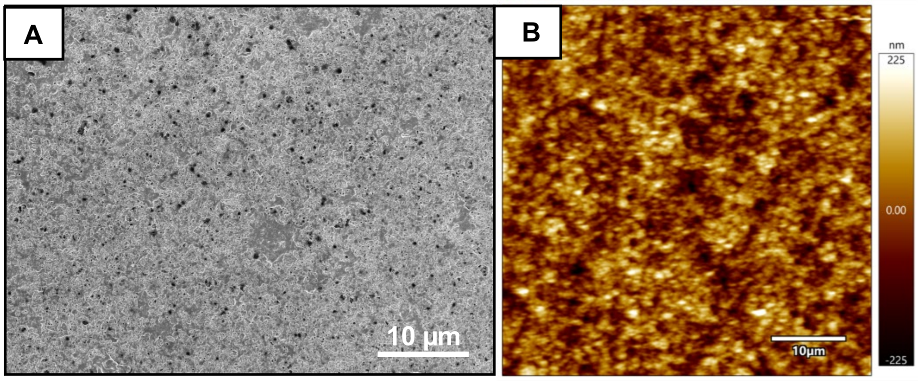

Fig. S1 FESEM and AFM images of physically abraded dip-coating. The successive application of adhesive tape peeling process exposed the interior of the coating.

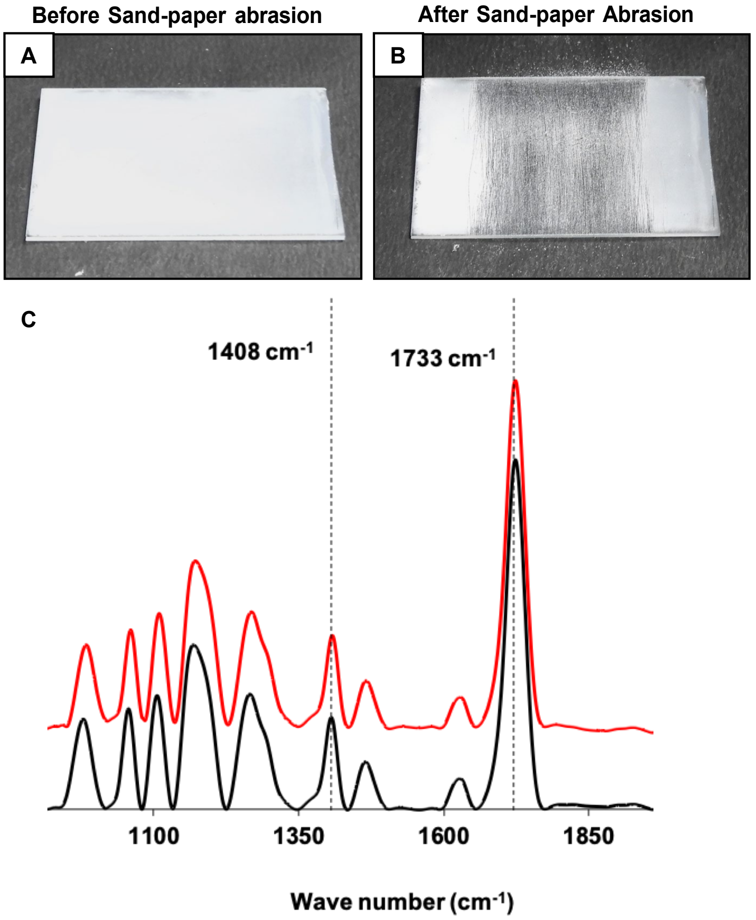

Fig. S2 A-B) Digital images of the chemically dip-coating before (A) and after (B) sand paper abrasion for 25 times with back and forth motion under 100 g load. C) ATR spectra of the chemically reactive coating before (black) and after (red) sand paper abrasion. ATR-FTIR signatures at 1408 cm^{-1} and 1733 cm^{-1} for carbonyl and vinylic C-H deformation respectively, revealed the presence of residual acrylate group.

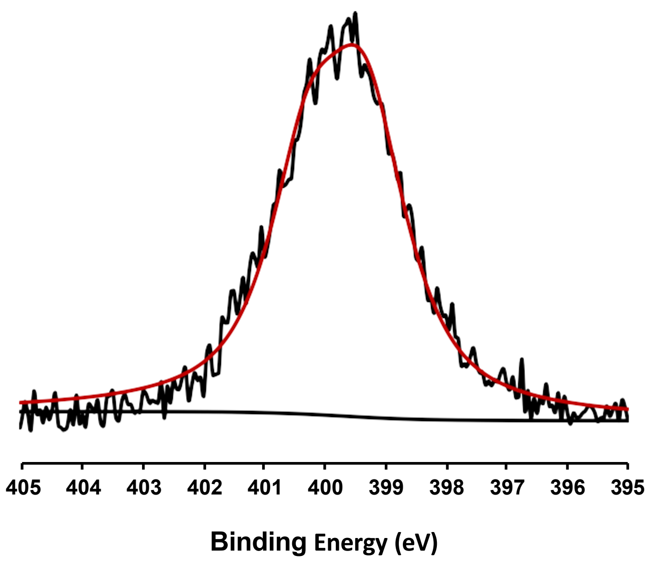

Fig. S3. XPS spectra of N 1s of the polymeric dip-coating, The XPS signature of dip-coating at ~ 400 eV revealed the presence of presence of residual amine

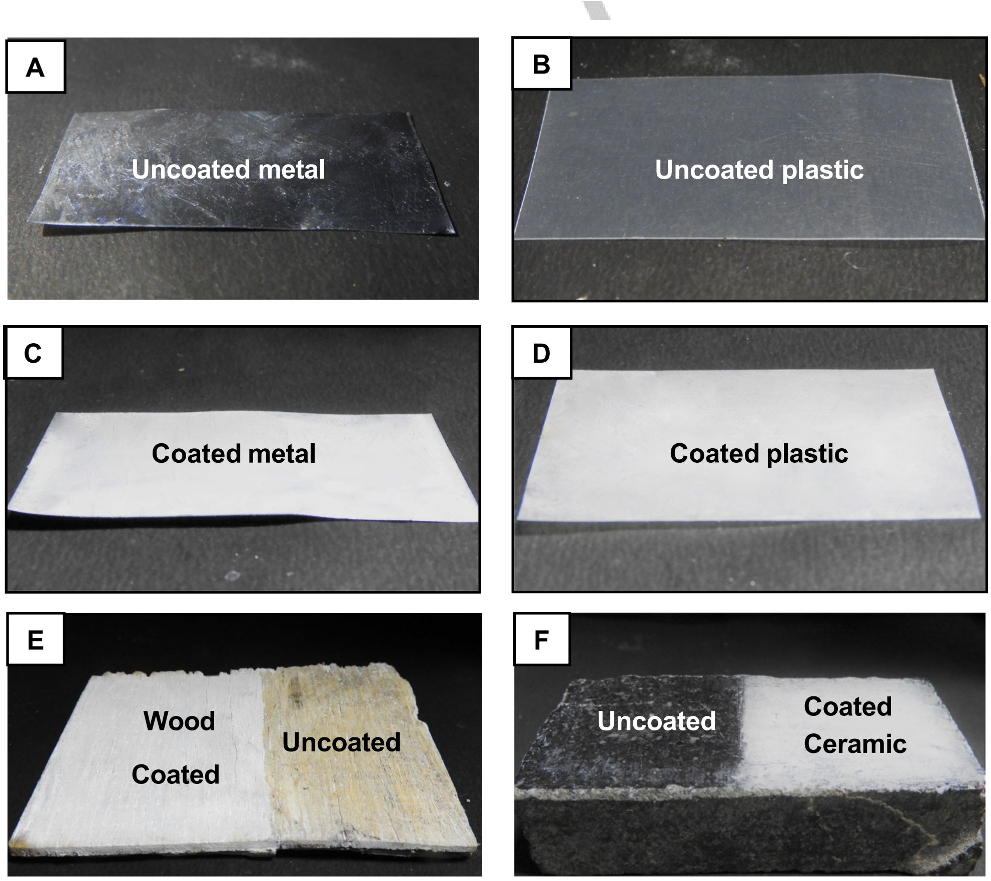

Fig. S4 A-F) Accounting the uncoated (A,B) and dip-coated metal (C), plastic (D), wood (E, left-side) and ceramic (F, right-side) substrates.

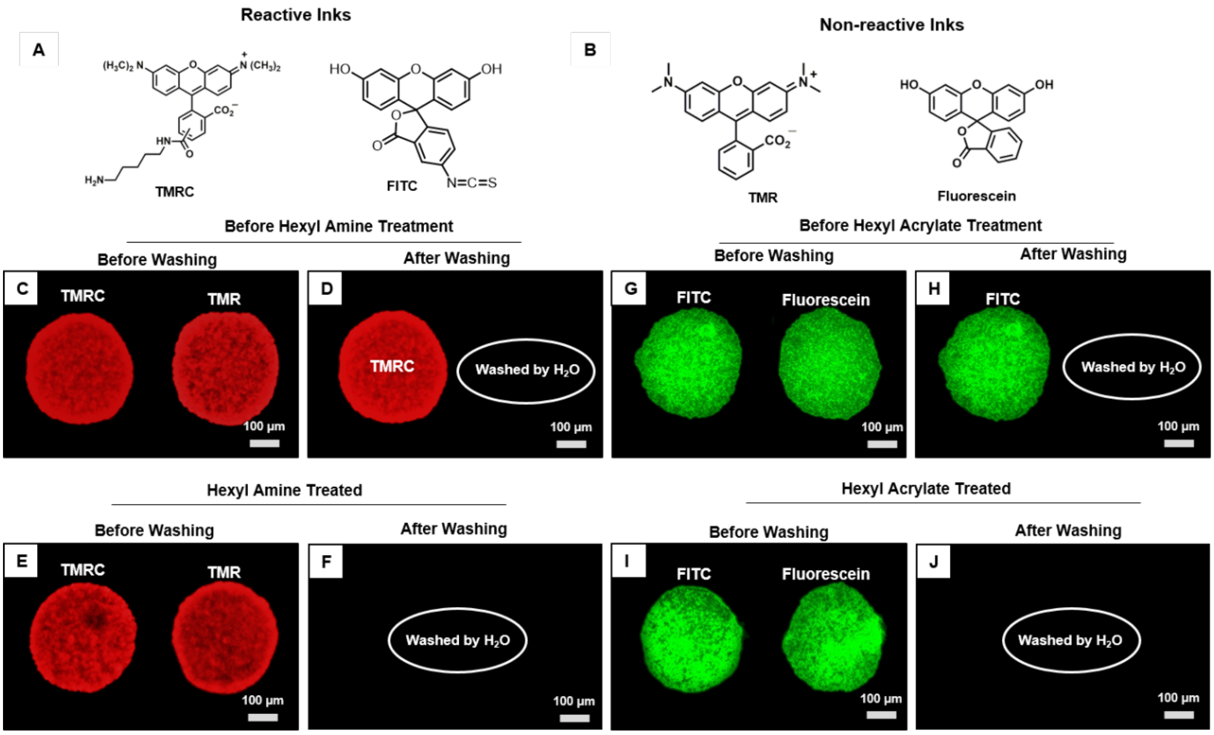

Fig. S5 Chemical structures of various (a) readily reactive (TMRC, FITC) and (b) non-reactive (TMR, fluorescein) fluorescent inks respectively. (c,d, g,h) Fluorescence microscopy images of dip-coating having the physically deposited spots of readily reactive (TMRC, FITC) and non-reactive (TMR/fluorescein) inks (g-j) before (c), (g) and after (d), (h) deionized water washing. After water washing non-reactive dyes disappeared (d), (h). (e), (f), (i), (j) Fluorescence images accounting the disappearance (f), (j) of both reactive and non-reactive inks after water washing from hexyl amine (e), (f) and hexyl acrylate (i), (j) treated dip-coatings. The lack of residual acrylate and amine in the post-modified (with hexylamine and hexylacrylate) dip-coating failed to covalently attach even chemically reactive coating.

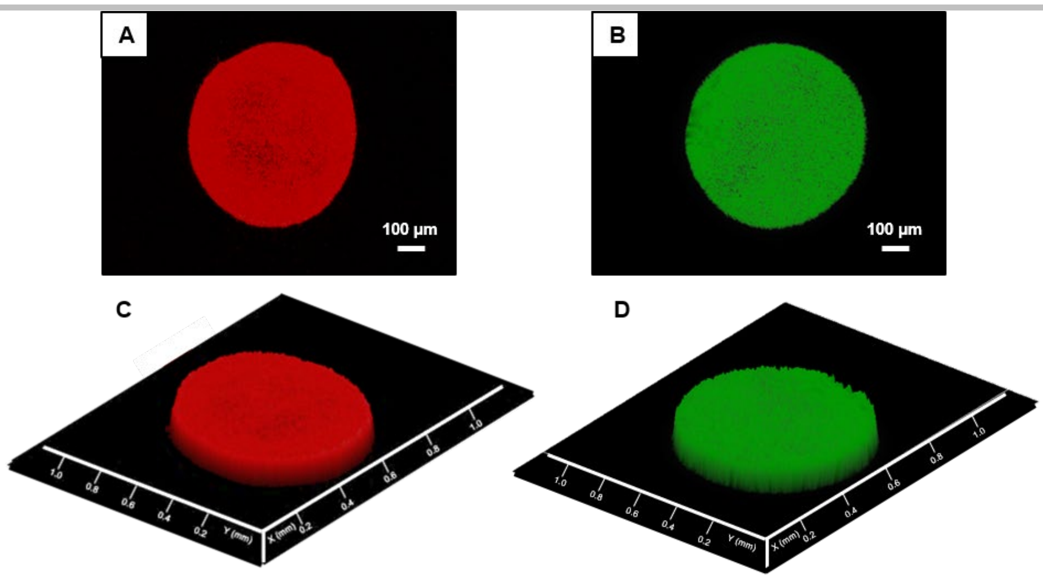

Fig. S6 Fluorescence confocal microscopy images of manually spotted ink droplets. (a) TMRC and (b) FITC inks. (c) and (d) show 3D representations of the images in (a) and (b) respectively.

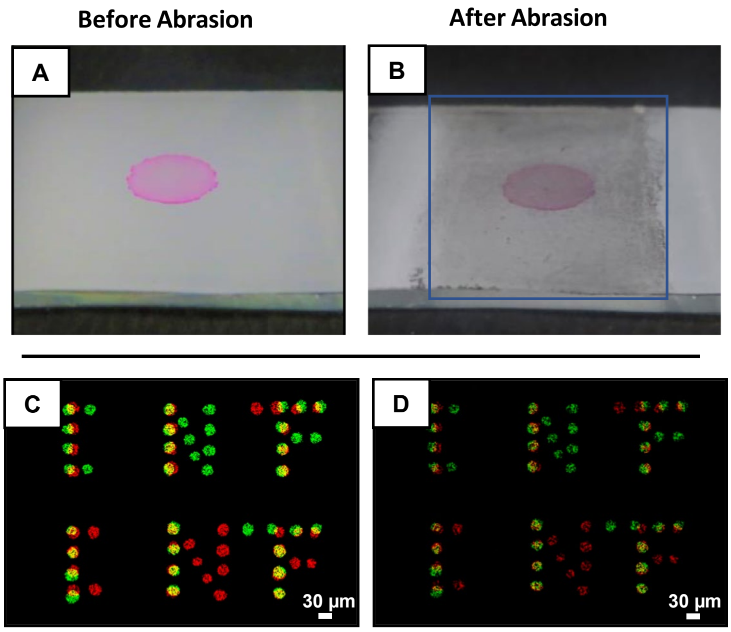

Fig. S7 Digital images of spatially selective modified (with aqueous droplet of red-dye) dip-coating before (A) and after (B) physical abrasion (adhesive tape peeling test). The physically abraded region is indicated by dotted box. The presence of faint red-stain in the physically abraded interface validated the bulk diffusion of used small molecule. In a separate experiment, (C-D) the merged fluorescence images of spatially selectively patterned dots of small molecules (TMRC & FITC) on the porous dip-coating before (C) and after (D) physical abrasion. The existence of fluorescence signals (red for TMRC & green for FITC) in physically abraded interface reconfirmed the bulk diffusion of the used small molecules.

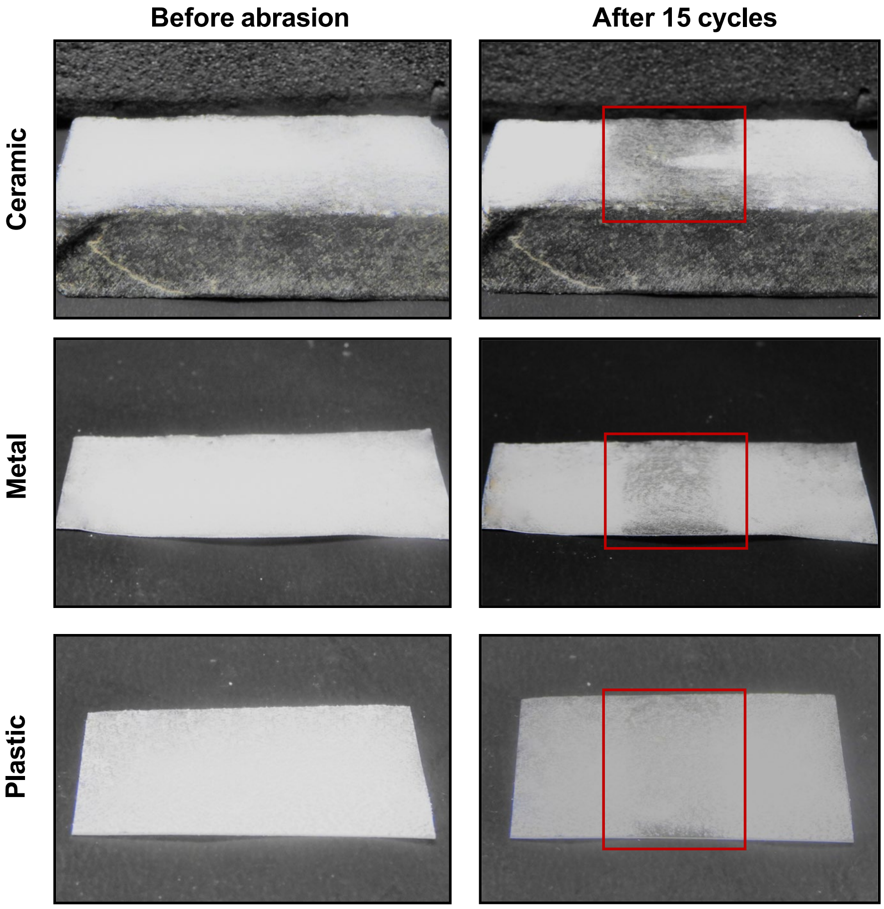

Fig. S8. Digital Images of coated ceramic, metal and plastic before and after performing adhesive tape peeling test for 15 times. The red box indicated the physically abraded area. Even after repetitive application of adhesive tape peeling test, a complete peeling of the deposited coating is not noted.

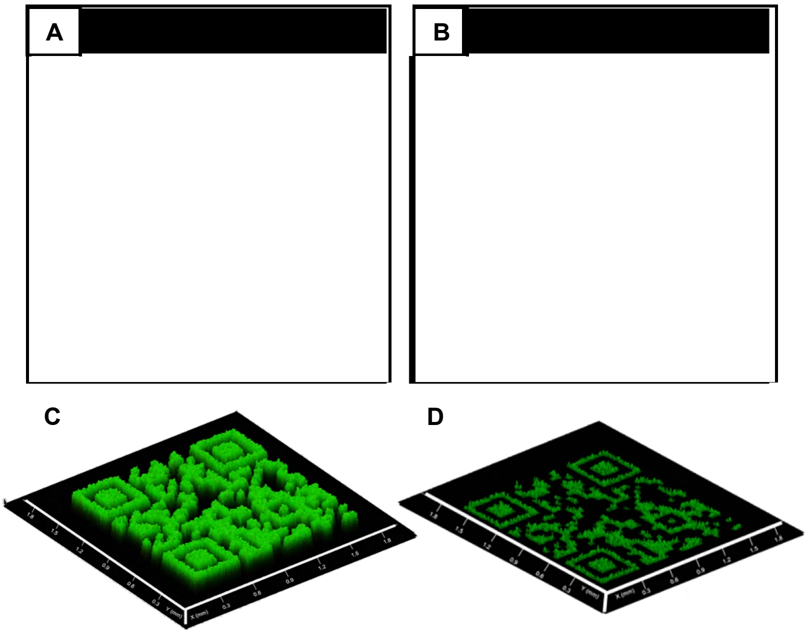

Fig. S9 A-D) Fluorescence microscopy images (A-B) and confocal (Z-stacked, C-D) image of an inkjet-printed QR code before (a) and after (b) physical abrasion. Image conditions were kept same to clearly show the impact of abrasion to the pattern by preserving the dynamic range of the fluorescence signal in the image. A contrast-enhanced version enabling direct readout also of the abraded pattern is shown in Figure S4.

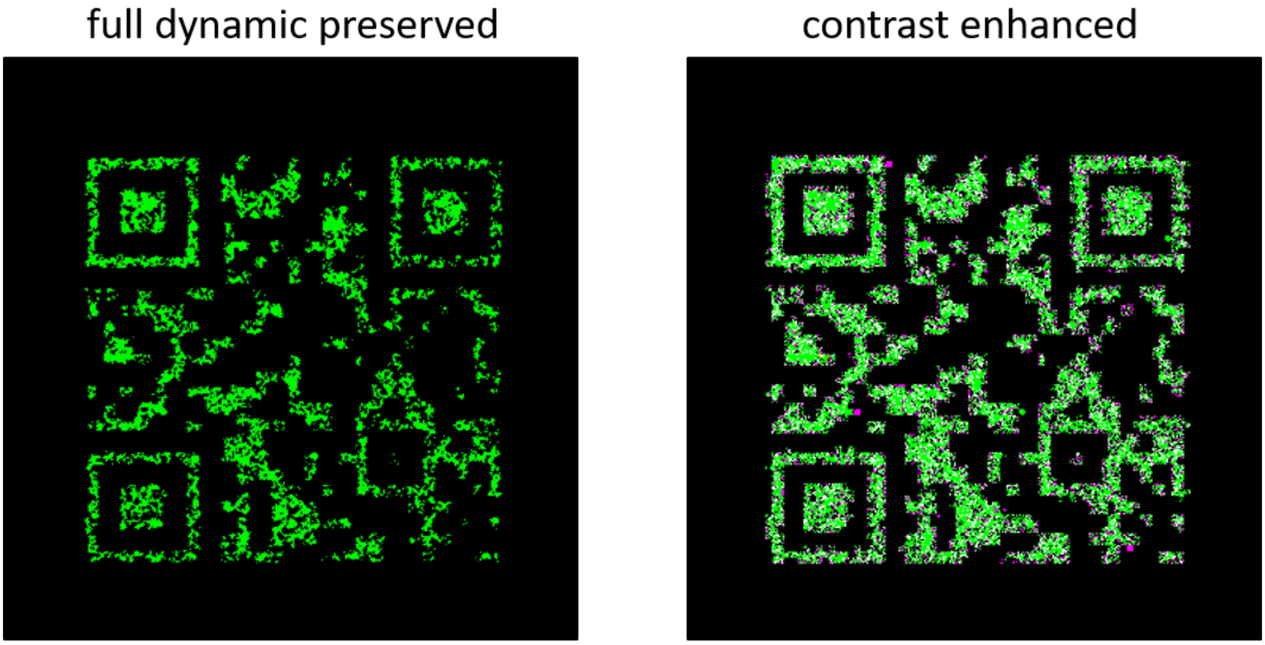

Fig. S10 Fluorescence microscopy images of QR code pattern after abrasion. Left in exposure conditions optimal for preserving full dynamic range in image and right a simply contrast enhanced version that allows direct readout of the code.

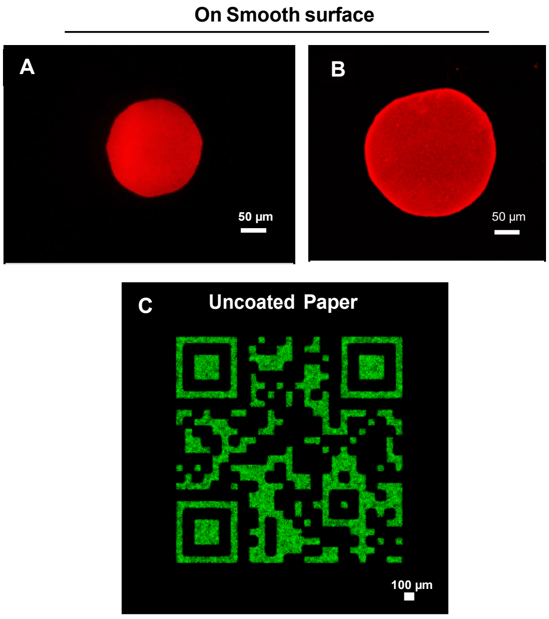

Fig. S11 Fluorescence microscopy images depicting the featureless edges of the same inks on (a), (b) smooth interfaces and (c) on bare paper.

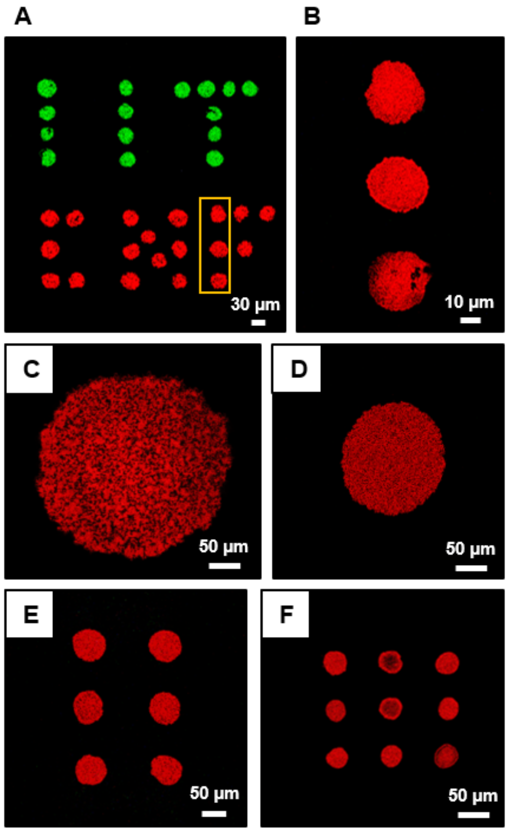

Fig. S12 A Fluorescence microscopy images of the dip-coating showing microscopic features of FITC and TMRC inks obtained by microchannel cantilever spotting (µCS). B) Magnified florescence image of the three spots of TMRC that indicated with yellow box in (A). (C-F) Florescence images accounting the edge effect of the spotted ink (TMRC) on the chemically reactive dip-coating in varying feature size.

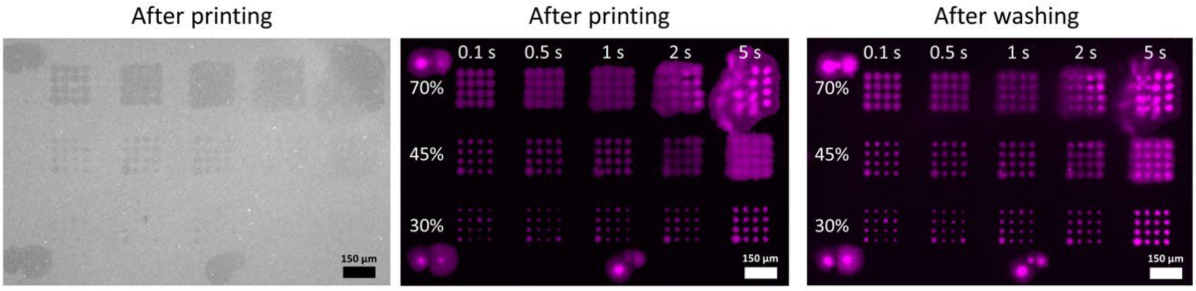

Fig. S13 Test-patterns for influence of dwell time and relative humidity on the µCS process. The humidity and dwell time were systematically varied during the spotting process as denoted in the florescence images.

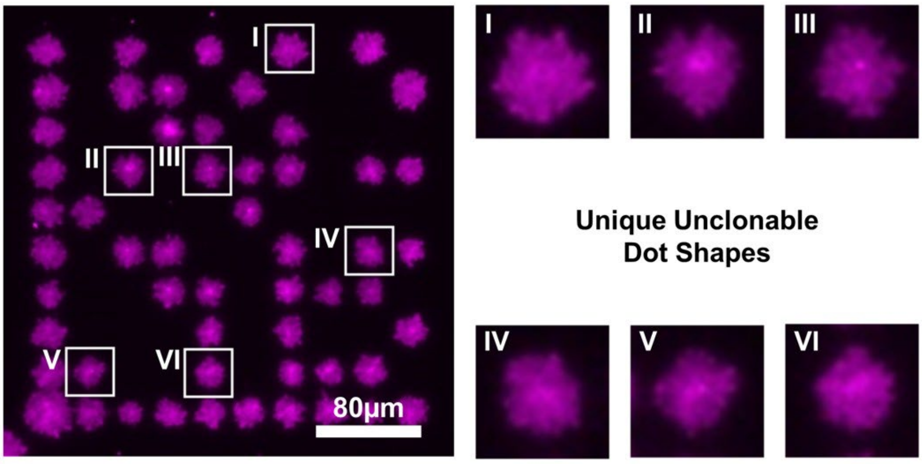

Fig. S14 A fluorescence image of a DataMatrix code exposed for optimal visibility of the feature borders. 6 selected features of the DataMatrix are enlarged on the right, to exemplify the unclonable dot shapes.

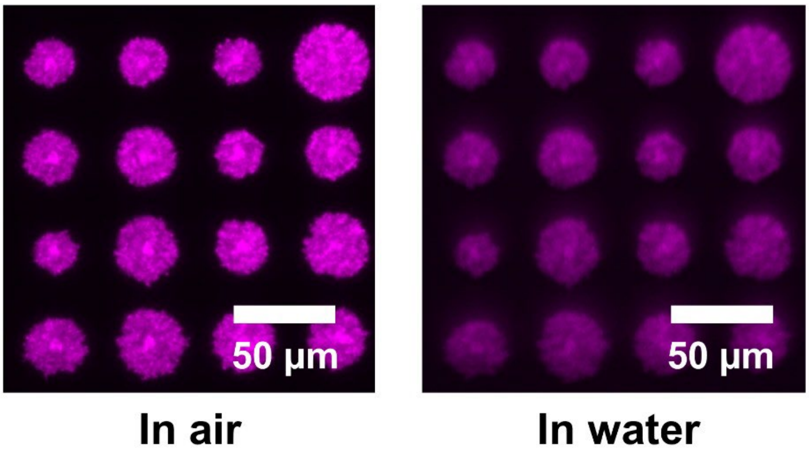

Fig. S15 Fluorescence images of a 4×4 dot array in air (left) and in water (right). While the intensity and sharpness of the optical images is slightly reduced due to observing through the water film, no substantial change besides this optical effect is noticable, implying the stability of the printed pattern as well the coating itself under humidity and liquid water.
